# Bioinformatic Tools Identify Chromosome-Specific DNA Probes and Facilitate Risk Assessment by Detecting Aneusomies in Extra-embryonic Tissues

**DOI:** 10.2174/138920212802510510

**Published:** 2012-09

**Authors:** Hui Zeng, Jingly F Weier, Mei Wang, Haig J Kassabian, Aris A Polyzos, Adolf Baumgartner, Benjamin O’Brien, Heinz-Ulli G Weier

**Affiliations:** 1 Department of Cancer & DNA Damage Responses, Life Sciences Division, University of California, Lawrence Berkeley National Laboratory, Berkeley, CA, USA; 2 Clinical Laboratories, University of California (UC), San Francisco, CA, USA; 3 Department of Diabetes, Beckman Research Institute of the City of Hope, Duarte, CA; 4 Department of Paediatric Cardiology, Herzzentrum, University of Leipzig, Leipzig, Germany; 5 William Harvey Research Institute, Queen Mary University, London, UK

**Keywords:** Aneusomy, Gestation, Cytotrophoblast, Fetal-maternal Interface, Bioinformatics, DNA Probes, Bacterial artificial chromosomes, Fluorescence in situ hybridization (FISH).

## Abstract

Despite their non-diseased nature, healthy human tissues may show a surprisingly large fraction of aneusomic or aneuploid cells. We have shown previously that hybridization of three to six non-isotopically labeled, chromosome-specific DNA probes reveals different proportions of aneuploid cells in individual compartments of the human placenta and the uterine wall. Using fluorescence in situ hybridization, we found that human invasive cytotrophoblasts isolated from anchoring villi or the uterine wall had gained individual chromosomes. Chromosome losses in placental or uterine tissues, on the other hand, were detected infrequently. A more thorough numerical analysis of all possible aneusomies occurring in these tissues and the investigation of their spatial as well as temporal distribution would further our understanding of the underlying biology, but it is hampered by the high cost of and limited access to DNA probes. Furthermore, multiplexing assays are difficult to set up with commercially available probes due to limited choices of probe labels. Many laboratories therefore attempt to develop their own DNA probe sets, often duplicating cloning and screening efforts underway elsewhere. In this review, we discuss the conventional approaches to the preparation of chromosome-specific DNA probes followed by a description of our approach using state-of-the-art bioinformatics and molecular biology tools for probe identification and manufacture. Novel probes that target gonosomes as well as two autosomes are presented as examples of rapid and inexpensive preparation of highly specific DNA probes for applications in placenta research and perinatal diagnostics.

## INTRODUCTION

The human placenta is a vital organ anchoring the fetus to the mother via the uterus and providing an interface for the transport of nutrients, gases and waste. The overwhelming number of chromosomal studies of the placenta has been performed on cells biopsied from floating villi, which were cultured for several days to obtain metaphase spreads for conventional chromosome banding analysis. We decided to perform investigations on uncultured interphase cells using fluorescence in situ hybridization (FISH), since cell viability or proliferation are minor concerns when using FISH [1-5]. Probes for our initial studies of aneuploidy in extra-embryonic tissues were obtained from a commercial source (Abbott, Des Moines, IL) [6, 7]. Probe sets were comprised of three to four chromosome enumerator probes (CEPs) targeting chromosome types, X, Y, 16 or 18, or locus-specific probes (LSPs) for chromosome 13 or 21 [7]. Studying the chromosomal make-up of cells in different compartments of anchoring villi and the uterine wall also referred to as ‘basal plate’, we found that the karyotypes of these extra-embryonic cells were mostly unrelated to the karyotype of the fetus [5, 7, 8]. The most common abnormality we have observed was a gestational age-related gain of chromosomes affecting invading cytotrophoblasts (iCTB’s) [7]. For a more comprehensive analysis and to be able to increase the number of chromosome types that can be scored simultaneously in a single FISH experiment, we had to develop our own custom sets of chromosome-specific DNA probes.

While the DNA probe development efforts described in the present communication were prompted by the need to develop a novel probe set for more comprehensive cytogenetic analyses of normal placental tissue compartments from uncomplicated pregnancies [6], DNA probes selected in a similar fashion are likely to find widespread application in investigations of unusual conditions such as spontaneous abortions [9, 10] or confined placental mosaicism (CPM) [11-14], the cytogenetic analysis of human preimplantation embryos [15-21], perinatal analysis [22-24], tumor research and diagnosis [1-5, 25-27] as well as radiobiological or environmental studies [28-40]. Thus, the description of our probe selection approach combining bioinformatics tools for data mining of genomic databases with deeply redundant recombinant DNA clone libraries, which follows the brief review of the more conventional techniques for DNA probe selection, may provide useful information for a diverse group of researchers in the life sciences and enable the average research lab to prepare chromosome-specific custom DNA probes at a very affordable cost.

### Selection of DNA Target Sequences and Preparation of Non-Isotopically Labeled DNA Probes for FISH

Briefly, successful cytogenetic analysis by FISH is based on the formation of stable hybrids between the DNA targets inside cell nuclei or metaphase chromosomes and the labeled DNA probes molecules provided by the investigator [41]. The DNA probes can either be marked by a fluorochrome, which can then be detected by eye or a camera attached to a fluorescence microscope, or by a non-fluorescent, non-isotopical hapten, most often biotin, digoxigenin or dinitrophenol, which is detected by a fluorescent moiety such as a fluorochrome-labeled avidin or antibody. Different probe types are available to suit particular applications: whole chromosome painting probes allow the delineation of inter-chromosomal translocations in metaphase spreads [37, 42, 43], while intra-chromosomal rearrangements are detected in metaphase or interphase cells with chromosome band-specific probes [44-47]. In addition, there are DNA probes that target somewhat smaller, gene- or locus-specific regions [34, 48-52].

While the FISH technology found widespread application in research laboratories around the world, its acceptance in clinical settings is still hampered by a limited selection of commercially available, U.S. Food and Drug Administration (FDA)-approved tests and the typically labor-intensive, costly nature of producing DNA probes that perform well in multiplexed assays [53]. While FDA approval may be required for all diagnostic probes that are shipped across state borders in the U.S., the in-house preparation of DNA probes might lead to significant cost savings in research laboratories. Our laboratories have a long-standing track record of production of novel DNA probes and innovative cytogenetic assays, many of which have found their way into contemporary cancer research or preimplantation genetic diagnosis (PGD) analysis [16, 43, 45, 47, 48, 50, 54-60]. To facilitate the distribution of molecular cytogenetic assays and make DNA probes as well as multiplex FISH tests available to the less experienced laboratory, we have undertaken probe production pilot studies which take advantage of the vast resources generated in the course of the Human Genome Project such as physical maps and recombinant DNA libraries. 

Our initial studies focused on the preparation of novel DNA probes for chromosome scoring or ‘enumeration’ in interphase cell nuclei and metaphase spreads, since these seem to remain the most common applications in research and the clinical settings [53, 61]. The vast majority of these CEPs target highly reiterated, tandemly-repeated DNA sequences in order to bind many copies of a rather small probe sequence to a tightly localized area or volume. Different ways of isolating and purifying such DNA probes exist [25, 54, 59, 60, 62-66]. 

Briefly, up until the 1980’s, satellite DNA sequences were enriched, isolated and characterized by a cumbersome, labor-intensive workflow which involved either density gradient centrifugation or timed reassociation of single stranded, thermally denatured DNA followed by enzymatic digestion of single stranded DNA by exonucleases. This was followed by molecular cloning, library screening, clone characterization and DNA sequencing which made this a rather costly enterprise [67-69]. The use of endonucleases to break up large tandemly repeated DNA clusters facilitated the hunt for chromosome-specific heterochromatic, satellite DNA, expedited the cloning-characterization steps and lead to major progress in the identification of chromosome-specific high order tandem repeats [62, 70-74].

**Fig. (1):**
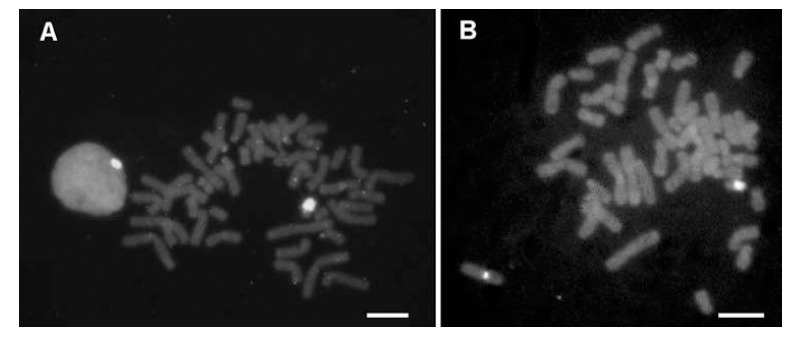
**In situ hybridization of cloned, chromosome-specific PCR products. A)** Biotinylated DNA prepared from PCR products with chromosome Y -specific oligonucleotide primers bind specifically to the heterochromatic region of the human Y chromosome. **B)** The Y-specific probe shown in **A)** can be combined with a biotinylated probe for the smaller tandemly repeated DNA cluster at the centromeric region of the X-chromosome. Bound probes were detected with avidin-FITC (green fluorescence) on ethidium bromide (red fluorescence) stained chromosomes, here shown as grey scale images. (Bars = 10 µm).

The breakthrough in the isolation of chromosome-specific DNA polynucleotides and preparation of DNA probes for FISH came with the application of DNA amplification using the polymerase chain reaction (PCR) in the late 1980’s: chromosome-specific sequences could be extracted on-line from larger, high order tandem repeats of satellite DNA to define the PCR primer sequences and amplify a specific fragment from genomic DNA [54] (Fig. **1A**).

In a variation of this scheme, chromosome-specific sequences could be amplified with consensus PCR primers from template DNA which provided limited sequence variety, such as flow-sorted human or mouse chromosomes [25, 75] (Fig. **1B**). In general, DNA probes generated this way still represented a pool of diverse sequences and molecular cloning was required to isolate the highly specific, informative probes [25].

It wasn’t until the completion of a first draft of the human genome sequence when new sets of genomic tools became available that would revolutionize the ways individual investigators analyze the human genome in the 1990’s and onwards often using no more than their personal computer and an on-line connection to publicly available databases. Large insert, recombinant DNA libraries such as YAC [76, 77], P1 [78, 79] or BAC [66, 80, 81] libraries had been constructed and characterized, clones had been end-sequenced and placed on the larger physical maps by basic sequence alignment procedures [82].

The work of Baumgartner *et al.* (2006) [65] showed that a combination of database searches (to identify BAC clones rich in satellite content) in combination with *in vitro* DNA amplification can expedite the preparation of chromosome-specific DNA probes. However, this approach still requires some *a priori* knowledge of the target sequence to specify the PCR primers [65].

**Fig. (2):**
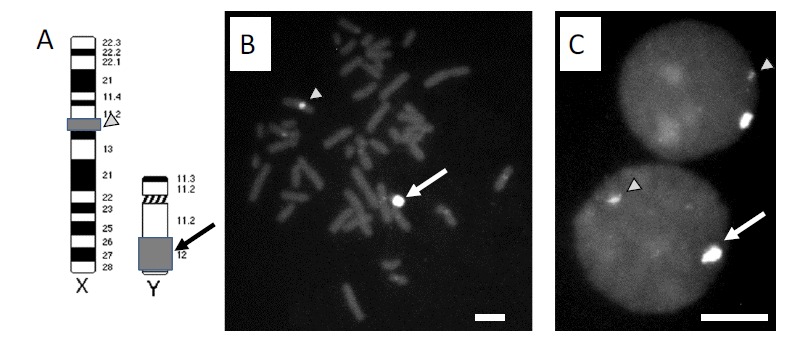
**In situ hybridization analysis of DNA probes prepared from BAC clones.** The BAC clones RP11-294C12 and RP11-242E13 hybridized to metaphase spreads prepared from short term cultures of human lymphocytes showed specific hybridization to the target regions on the X (arrowhead) and Y (arrow) chromosome, respectively. **A)** Schematic representation of the FISH target regions on the X and Y chromosome. **B)** Hybridization of both probes to metaphase chromosomes. **C)** Hybridization signals in diploid interphase cell nuclei. (Bars = 10 µm).

We recently demonstrated that publicly available on-line databases can be analyzed using a suite of simple bioinformatics tools to identify chromosome-specific BAC clones [60]. Specifically, we used our proprietary information of a Y chromosome-specific sequence [83-85] and a DNA sequence alignment program (BLAST) [82] to identify BAC clone RP11-243E13 as a potential DNA probe. Using the Genome Browser program at the UC Santa Cruz (UCSC) Genome Center web site (genome.ucsc.edu), we then identified a BAC clone mapped to the satellite containing centromeric heterochromatin on the human X chromosome (BAC RP11-294C12) [60]. Probes prepared from these two BAC clones showed an impressive better-than-expected performance in FISH experiments by displaying strong, highly specific FISH signals localized exclusively to the target chromosomes (Fig. **2**).

### Probe Preparation and Fluorescence in Situ Hybridization (FISH) of BAC-derived DNA Probes

The procedures used for hybridization of BAC-derived DNA probes follow pretty much the published procedures for oligonucleotide, plasmid or P1-derived DNA probes [50, 86, 87]. In typical experiments, the BAC DNAs are extracted from overnight cultures following an alkaline lysis protocol [88] or using a BAC DNA miniprep kit (Zymo Research; Irvine, CA). The DNAs are confirmed on a 1% agarose gel and quantitated spectrophotometrically. Probe DNAs are labeled with biotin-14-dCTP or digoxigenin-11-dUTP (Roche; Indianapolis, IN) by random priming using a commercial kit (BioPrime Kit, Invitrogen; Carlsbad, CA). Slides of metaphase spreads of cells are made from short-term cultures of peripheral blood lymphocytes from a karyotypically normal male following published procedures [35].

The slides (metaphase cells, interphase cell nuclei or slides carrying deparaffinized tissue section) are denatured in 70% formamide at 70 °C, dehydrated and overlaid with a hybridization cocktail containing 20-50 ng of denatured probe DNA in buffer containing 10% dextran sulfate and 50-55 % formamide. Following overnight incubation at 37°C (48 or more hours for deparaffinized tissue sections), slides are washed to remove excess probes and incubated with a fluorochrome-conjugated avidin or corresponding antibodies as required [59, 66, 89]. Finally, the slides are mounted with 4,6-diamino-2-phenylindole (DAPI) (0.1µg/ml) in antifade solution coverslipped and imaged on a fluorescence microscope.

### BAC-Derived DNA Repeat Probes for Autosomal Targets

We were also interested in whether this concept of knowledge-based probe selection can be extended to probes for human autosomes. In our 2006 paper [65], we had proposed a satellite-rich BAC clone, RP11-469P16, as template for a PCR based probe generation scheme. The UCSC Human Genome Browser at genome.ucsc.edu indicates the presence of a long interspersed repeated DNA sequence (LINE) in the BAC insert, which may lead to undesirable cross-hybridization since LINEs are not chromosome-specific, but exist in thousands of copies across the human genome. 

**Fig. (3):**
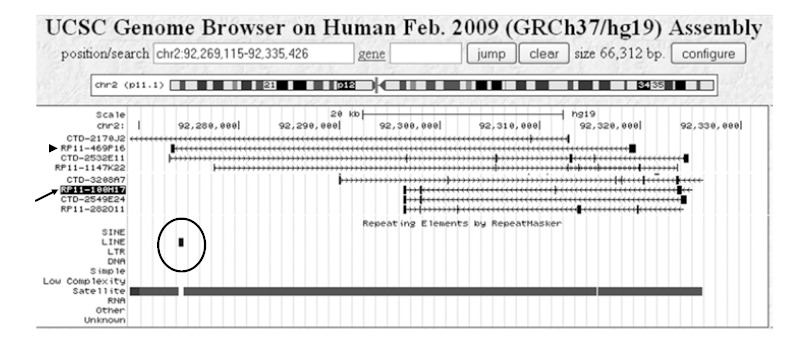
Screen dump of the UCSC Genome Browser (GoldenPath) display showing the BAC ends mapped to the targeted region of chromosome 2: 92,269,115 bp to 92,335,426 bp. The alignment BAC clone end sequences with the draft sequence of the human genome places BAC RP11-100H17 (arrow) in a region comprised entirely of satellite DNA, while BAC RP11-469P16 (arrowhead) is predicted to contain a cluster of long interspersed repeated DNA sequences (LINEs)(circled).

According to information provided on the UCSC Genome browser web site, a BAC insert typically consists of 25-350 kb of DNA. During the early phase of a sequencing project, it is common to sequence a single read (approximately 500 bases) at each end of each BAC from a large library. Later on in the project, these BAC end reads are mapped *in silico* to the genome draft sequence. Tracks in the genome browser as shown in Fig. **3** show these mappings in cases where both paired ends could be mapped within. A valid pair of BAC end sequences must be at least 25 kb but no more than 350 kb away from each other. The orientation of the first BAC end sequence must be "+" and the orientation of the second BAC end sequence must be "-". BAC end sequences are placed on the assembled sequence using Jim Kent's blat program [90]. Tracks can be used for determining which BAC contains a given gene or DNA repeat clusters using the ‘RepeatMasker’ program (www.repeatmasker.org). Please note that for the heterochromatic regions, there has been almost no clone validation in place to ensure that the predicted size or location of the BAC probe is correct.

**Fig. (4):**
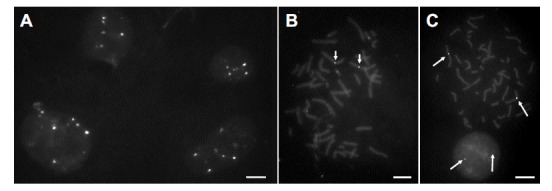
**FISH performance of BAC-derived DNA probes targeting the centromeric heterochromatin of chromosome 2. A-B)** A DNA probe prepared from BAC clone RP11-469P16 shows multiple signals in normal interphase cell nuclei (**A**) or on metaphase spreads (**B**). Arrows in **B**) point at the target region on chromosome 2. **C**) A DNA probe prepared from BAC clone RP11-100H17 binds exclusively to the chromosome 2-specific target region (arrows). (Bars = 10 µm).

When using a DNA probe prepared from BAC RP11-469P16, FISH results showed cross-hybridization to multiple chromosomes other than chromosome 2 (Fig. **4A**, **B**). However, a DNA probe prepared from BAC clone RP11-100H17 (Fig. **3**, arrow), which is expected to bind ~20 kb proximal of RP11-469P16 on the short arm of human chromosome 2, gave strong, highly specific FISH signals on interphase and metaphase cells (Fig. **4C**). This can be attributed to the lack of interspersed non-chromosome specific DNA repeats in the insert of BAC RP11-100H17 as well as it’s composition of DNA tandem repeat units of entirely chromosome 2-specific satellite DNA.

**Fig. (5):**
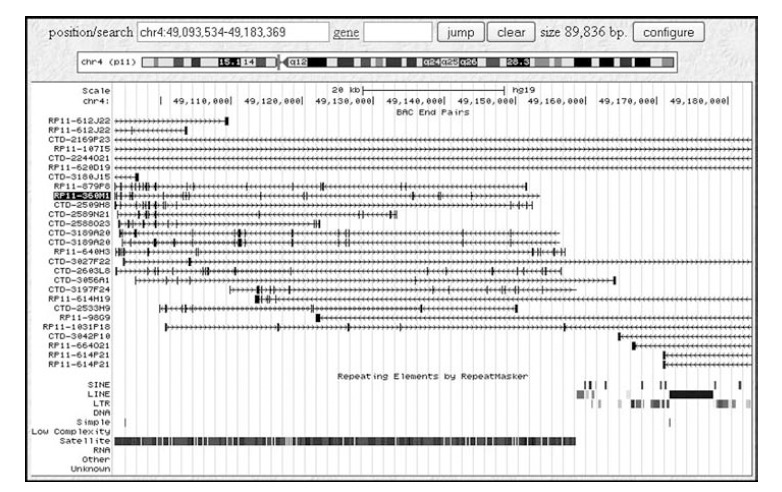
Screen dump of the UCSC Genome Browser display showing the BAC ends mapped to the region chromosome 4: 49,093,534 bp to 49,183,369 bp. The alignment BAC clone end sequences with the draft sequence of the human genome places BAC RP11-360M1 (high-lighted) in a region comprised almost entirely of satellite DNA and completely free of interspersed repeated DNA sequences such as short interspersed repeats (SINEs) or LINEs.

Since inserts of BAC clones that contain satellite DNA, but no short or long interspersed repeated DNA sequences (SINEs, LINEs) appear to render a high signal-to-noise ratio and strong chromosome specific signals which can easily be scored by eye using a microscope, we prepared a SINE-/LINE-free DNA probe for the short arm of chromosome 4, band p11. The BAC RP11-360M1 carries an insert of an estimated 59846 bp, which is rich in tandemly-repeated satellite DNA repeats, but free of interspersed repeat DNA (Fig. **5**).

**Fig. (6):**
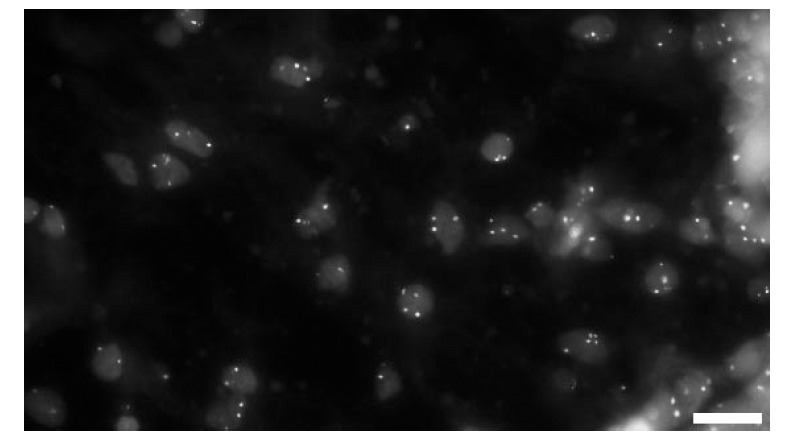
**FISH analysis of placental tissue sections.** Probes prepared from BAC clones RP11-294C12 (confirmed target is chromosome X) and RP11-360M1 (supposed to bind to chromosome 4) give compact, easy-to-score hybridization signals on formalin-fixed, paraffin-embedded tissue sections. The red or green signals from the original image together with the DAPI counterstain are overlayed in this gray-scale image. (Bars = 10 mm).

In situ hybridization of the chromosome 4-specific DNA probe prepared from BAC RP11-360M1 in combination with a differently lebeled probe (BAC RP11-294C12) for the centromeric region of the X chromosome to deparaffinized human placental tissue section showed excellent probe performance, i.e., strong and highly specific DNA signals with were easily scored (Fig. **6**).

## CONCLUDING REMARKS

Molecular cytogenetic analyses using FISH have provided major contributions to our understanding of disease processes including tumorigenesis, cancer progression and metastasis, but also to the existence of aneuploid cell populations or cohorts in seemingly normal tissues [5, 61, 91-96].

For example, with an incidence of one in every 5-6 clinically recognized pregnancies, spontaneous abortions (SABs) during the first trimester are the most frequent pregnancy complication in women [9]. Causes of SABs have been identified as chromosomal abnormalities, uterine defects, immunological problems, hormonal imbalance and infections [2-6]. While more than half of all first trimester SABs are associated with chromosomal abnormalities, nearly 40% remain unexplained [6]. With no apparent association between placental villous morphology and fetal chromosomal abnormalities, SABs with either euploid or aneuploid conceptuses demonstrated incomplete cytotrophoblast (CTB) differentiation and compromised invasion [7-9]. These observations prompted our studies of the chromosomal make-up of extra-embryonic cells at materno-embryonic and fetal-maternal interfaces, i.e., the human placenta and the uterine wall. However, as mentioned in the introduction the application of DNA probes described in this review is not limited to investigations of fetal or extra-embryonic tissues.

The novel database mining approach to DNA probe selection described here is a fast and inexpensive solution to the problems of ‘probe bottlenecks’ in clinical research. Mapping information for BAC clones is publicly available from UCSC or the National Center for Biomedical Information (NCBI)/National Institute of Health, USA, different libraries outside the US, such as the Wellcome Trust Sanger Institute, Hinxton, UK, or the Resources for Molecular Cytogenetics, Dipartimento di Genetica e Microbiologia, Universita' di Bari, Bari, Italy, as well as several commercial sources are available to purchase these clones. The BAC-derived satellite DNA probes also seem to out perform most of the chromosome enumerator probes that are presently in use in research and clinical laboratories. In summary, the procedures described in the present communication allow a laboratory with typical, non-specialist equipment to prepare chromosome-specific DNA probes in just a few days and thus represent the most efficient, rapid and cost-conscious approach to generation of chromosome-specific DNA probes for cytogenetic studies.

## DISCLAIMER

This document was prepared as an account of work sponsored by the United States Government. While this document is believed to contain correct information, neither the United States Government nor any agency thereof, nor The Regents of the University of California, nor any of their employees, makes any warranty, express or implied, or assumes any legal responsibility for the accuracy, completeness, or usefulness of any information, apparatus, product, or process disclosed, or represents that its use would not infringe privately owned rights. Reference herein to any specific commercial product, process, or service by its trade name, trademark, manufacturer, or otherwise, does not necessarily constitute or imply its endorsement, recommendation, or favoring by the United States Government or any agency thereof, or The Regents of the University of California. The views and opinions of authors expressed herein do not necessarily state or reflect those of the United States Government or any agency thereof, or The Regents of the University of California.

## ABBREVIATIONS

BAC: = Bacterial Artificial Chromosome*
CEP: = Chromosome Enumerator Probe
CPM: = Confined Placental Mosaicism
DAPI: = 4,6-Diamino-2-phenylindole
iCTB: = Invasive Cytotrophoblast
LINE: = Long Interspersed DNA Repeat
LSP: = Locus-Specific Probe
SAB: = Spontaneous Abortions
SINE: = Short Interspersed DNA Repeat
UCSC: = University of California Santa Cruz
YAC: = Yeast Artificial Chromosome
PGD: = Preimplantation Genetic Diagnosis

## References

[1] Tkachuk DC, Pinkel D, Kuo WL, Weier HU, Gray JW (1991). Clinical applications of fluorescence in situ hybridization. Genet. Anal. Tech. Appl.

[2] Matsuta M, Matsuta M, Kon S, Thompson C, LeBoit PE, Weier HU, Gray JW (1994). Interphase cytogenetics of melanocytic neoplasms: numerical aberrations of chromosomes can be detected in interphase nuclei using centromeric DNA probes. J. Cutan. Pathol.

[3] Zitzelsberger H, Szucs S, Weier HU, Lehmann L, Braselmann H, Enders S, Schilling A, Breul J, Hofler H, Bauchinger M (1994). Numerical abnormalities of chromosome 7 in human prostate cancer detected by fluorescence in situ hybridization (FISH) on paraffin-embedded tissue sections with centromere-specific DNA probes. J. Pathol.

[4] Zitzelsberger H, Szucs S, Robens E, Weier HU, Hoefler H, Bauchinger M (1996). Combined cytogenetic and molecular genetic analyses of fifty-nine untreated human prostate carcinomas. Cancer Genet. Cytogenet.

[5] Weier JF, Weier HU, Jung CJ, Gormley M, Zhou Y, Chu LW, Genbacev O, Wright AA, Fisher SJ (2005). Human cytotrophoblasts acquire aneuploidies as they differentiate to an invasive phenotype. Dev. Biol.

[6] Weier JF, Ferlatte C, Baumgartner A, Jung CJ, Nguyen HN, Chu LW, Pedersen RA, Fisher SJ, Weier HU (2006). Molecular cytogenetic studies towards the full karyotype analysis of human blastocysts and cytotrophoblasts. Cytogenet. Genome Res.

[7] Weier JF, Ferlatte C, Weier HU (2010). Somatic genomic variations in extra-embryonic tissues. Curr. Genomics.

[8] Wright A, Zhou Y, Weier JF, Caceres E, Kapidzic M, Tabata T, Kahn M, Nash C, Fisher SJ (2004). Trisomy 21 is associated with variable defects in cytotrophoblast differentiation along the invasive pathway. Am. J. Med. Genet.

[9] Zinaman MJ, Clegg ED, Brown CC, O'Connor J, Selevan SG (1996). Estimates of human fertility and pregnancy loss. Fertil. Steril.

[10] Hassold T (1986). Chromosome abnormalities in human reproductive wastage. Trends Genet.

[11] Wolstenholme J, Rooney DE, Davison EV (1994). Confined placental mosaicism, IUGR, and adverse pregnancy outcome: a controlled retrospective U.K. collaborative survey. Prenat. Diagn.

[12] Kalousek DK, Vekemans M (1996). Confined placental mosaicism. J. Med. Genet.

[13] Henderson KG, Shaw TE, Barrett IJ, Telenius AH, Wilson RD, Kalousek DK (1996). Distribution of mosaicism in human placentae. Hum. Genet.

[14] Kalousek DK (2000). Pathogenesis of chromosomal mosaicism and its effect on early human development. Am. J. Med. Genet.

[15] Munné S, Grifo J, Cohen J, Weier HU (1994). Chromosome abnormalities in human arrested preimplantation embryos: a multiple-probe FISH study. Am. j. Hum. Genet.

[16] Munné S, Weier HU, Grifo J, Cohen J (1994). Chromosome mosaicism in human embryos. Biol. Reprod.

[17] Benadiva CA, Kligman I, Munné S (1996). Aneuploidy 16 in human embryos increases significantly with maternal age. Fertil. Steril.

[18] Munné S, Magli C, Bahce M, Fung J, Legator M, Morrison L, Cohert J, Gianaroli L (1998). Preimplantation diagnosis of the aneuploidies most commonly found in spontaneous abortions and live births: XY, 13, 14, 15, 16, 18, 21, 22. Prenat. Diagn.

[19] Munné S (2002). Preimplantation genetic diagnosis of numerical and structural chromosome abnormalities. Reprod. Biomed. Online.

[20] Lathi RB, Westphal LM, Milki AA (2008). Aneuploidy in the miscarriages of infertile women and the potential benefit of preimplanation genetic diagnosis. Fertil. Steril.

[21] Fung J, Weier HU, Goldberg JD, Pedersen RA (2000). Multilocus genetic analysis of single interphase cells by spectral imaging. Hum. Genet.

[22] Ried T, Landes G, Dackowski W, Klinger K, Ward DC (1992). Multicolor fluorescence in situ hybridization for the simultaneous detection of probe sets for chromosomes 13, 18, 21, X and Y in uncultured amniotic fluid cells. Hum. Mol. Genet.

[23] Shaffer LG, Bui TH (2007). Molecular cytogenetic and rapid aneuploidy detection methods in prenatal diagnosis. Am. J. Med. Genet. C Semin. Med. Genet.

[24] Liehr T (2010). Cytogenetic contribution to uniparental disomy (UPD). Mol. Cytogenet.

[25] Weier HU, Kleine HD, Gray JW (1991). Labeling of the centromeric region on human chromosome 8 by in situ hybridization. Hum. Genet.

[26] Zitzelsberger H, Lehmann L, Hieber L, Weier HU, Janish C, Fung J, Negele T, Spelsberg F, Lengfelder E, Demidchik EP, Salassidis K, Kellerer AM, Werner M, Bauchinger M (1999). Cytogenetic changes in radiation-induced tumors of the thyroid. Cancer Res.

[27] Wlodarska I, Mecucci C, De Wolf-Peeters C, Verhoef G, Weier HU, Cassiman JJ, Van Den Berghe H (1994). "Jumping" translocation of 9q in a case of follicular lymphoma. Cancer Genet. Cytogenet.

[28] Wyrobek AJ, Robbins WA, Mehraein Y, Pinkel D, Weier HU (1994). Detection of sex chromosomal aneuploidies X-X, Y-Y, and X-Y in human sperm using two-chromosome fluorescence in situ hybridization. Am. J. Med. Genet.

[29] Robbins WA, Baulch JE, Moore D, Weier HU, Blakey D, Wyrobek AJ (1995). Three-probe fluorescence in situ hybridization to assess chromosome X, Y, and 8 aneuploidy in sperm of 14 men from two healthy groups: evidence for a paternal age effect on sperm aneuploidy. Reprod. Fertil. Dev.

[30] Robbins WA, Meistrich ML, Moore D, Hagemeister FB, Weier HU, Cassel MJ, Wilson G, Eskenazi B, Wyrobek AJ (1997). Chemotherapy induces transient sex chromosomal and autosomal aneuploidy in human sperm. Nat. Genet.

[31] Adler ID, Bishop J, Lowe X, Schmid TE, Schriever-Schwemmer G, Xu W, Wyrobek AJ (1996). Spontaneous rates of sex chromosomal aneuploidies in sperm and offspring of mice: a validation of the detection of aneuploid sperm by fluorescence in situ hybridization. Mutat. Res.

[32] Miller BM, Zitzelsberger HF, Weier HU, Adler ID (1991). Classification of micronuclei in murine erythrocytes: immunofluorescent staining using CREST antibodies compared to in situ hybridization with biotinylated gamma satellite DNA. Mutagenesis.

[33] Schmid TE, Xu W, Adler ID (1999). Detection of aneuploidy by multicolor FISH in mouse sperm after *in vivo* treatment with acrylamide, colchicine, diazepam or thiabendazole. Mutagenesis.

[34] Greulich KM, Kreja L, Heinze B, Rhein AP, Weier HG, Bruckner M, Fuchs P, Molls M (2000). Rapid detection of radiation-induced chromosomal aberrations in lymphocytes and hematopoietic progenitor cells by mFISH. Mutat. Res.

[35] Fung J, Weier HU, Pedersen RA, Zitzelsberger H, B Rautenstrauss, T Liehr (2002). Spectral imaging
analysis of metaphase and interphase cells. FISH Technology.

[36] Hessel H, Mittermuller J, Zitzelsberger H, Weier HU, Bauchinger M (1996). Combined immunophenotyping and FISH with sex chromosome-specific DNA probes for the detection of chimerism in epidermal Langerhans cells after sex-mismatched bone marrow transplantation. Histochem. Cell Biol.

[37] Zitzelsberger H, Bruch J, Smida J, Hieber L, Peddie CM, Bryant PE, Riches AC, Fung J, Weier HU, Bauchinger M (2001). Clonal chromosomal aberrations in simian virus 40-transfected human thyroid cells and in derived tumors developed after *in vitro* irradiation. Int. J. Cancer.

[38] Baumgartner A, Schmid TE, Schuetz CG, Adler ID (2001). Detection of aneuploidy in rodent and human sperm by multicolor FISH after chronic exposure to diazepam. Mutat. Res.

[39] Weier HU, Greulich-Bode KM, Ito Y, Lersch RA, Fung J (2002). FISH in cancer diagnosis and prognostication: from cause to course of disease. Expert Rev. Mol. Diagn.

[40] Weier HU, Ito Y, Kwan J, Smida J, Weier JF, Hieber L, Lu CM, Lehmann L, Wang M, Kassabian HJ, Zeng H, O'Brien B (2011). Delineating Chromosomal Breakpoints in Radiation-Induced Papillary Thyroid Cancer. Genes (Basel).

[41] Lichter P, Ward DC (1990). Is non-isotopic in situ hybridization finally coming of age?. Nature.

[42] Weier HU, Lucas JN, Poggensee M, Segraves R, Pinkel D, Gray JW (1991). Two-color hybridization with high complexity chromosome-specific probes and a degenerate alpha satellite probe DNA allows unambiguous discrimination between symmetrical and asymmetrical translocations. Chromosoma.

[43] Weier H-U, Pinkel D, Gray JW, Meyers R A (1995). Whole-Chromosome Complementary
Probe Fluorescence Staining. Molecular Biology and Biotechnology.

[44] Liehr T, Weise A, Heller A, Starke H, Mrasek K, Kuechler A, Weier HU, Claussen U (2002). Multicolor chromosome banding (MCB) with YAC/BAC-based probes and region-specific microdissection DNA libraries. Cytogenet. Genome Res.

[45] Zitzelsberger H, O'Brien B, Weier HU, Rautenstrauss B, Liehr T (2002). Multicolor FISH techniques for the detection of inter- and intrachromosomal rearrangements. FISH Technology.

[46] Liehr T, Nietzel A, Starke H, Heller A, Weise A, Kuechler A, Senger G, Ebner S, Martin T, Stumm M, Wegner R, Tonnies H, Hoppe C, Claussen U, Von Eggeling F (2003). Characterization of Small Marker Chromosomes (SMC) by Recently Developed Molecular Cytogenetic Approaches. J. Assoc. Genet. Technol.

[47] O'Brien B, Jossart GH, Ito Y, Greulich-Bode KM, Weier JF, Munné S, Clark OH, Weier HUG (2010). ‘Chromosomal Rainbows’ detect oncogenic rearrangements of signaling molecules in thyroid tumors. The Open Cell Signaling Journal.

[48] Jossart GH, O'Brien B, Cheng JF, Tong Q, Jhiang SM, Duh Q, Clark OH, Weier HU (1996). A novel multicolor hybridization scheme applied to localization of a transcribed sequence (D10S170/H4) and deletion mapping in the thyroid cancer cell line TPC-1. Cytogenet. Cell Genet.

[49] Cassel MJ, Munné S, Fung J, Weier HU (1997). Carrier-specific break-point-spanning DNA probes: an approach to preimplantation genetic diagnosis in interphase cells. Hum. Reprod.

[50] Weier HU, Rhein AP, Shadravan F, Collins C, Polikoff D (1995). Rapid physical mapping of the human trk protooncogene (NTRK1) to human chromosome 1q21-q22 by P1 clone selection, fluorescence in situ hybridization (FISH), and computer-assisted microscopy. Genomics.

[51] Greulich-Bode KM, Wang M, Rhein AP, Weier JF, Weier HU (2008). Validation of DNA probes for molecular cytogenetics by mapping onto immobilized circular DNA. Mol. Cytogenet.

[52] Chen XN, Korenberg JR (2002). BAC resource for molecular cytogenetics. Methods Mol. Biol.

[53] Munné S, Howles CM, Wells D (2009). The role of preimplantation genetic diagnosis in diagnosing embryo aneuploidy. Curr. Opin. Obstet. Gynecol.

[54] Weier HU, Segraves R, Pinkel D, Gray JW (1990). Synthesis of Y chromosome-specific labeled DNA probes by *in vitro* DNA amplification. J. Histochem. Cytochem.

[55] Weier HU, Polikoff D, Fawcett JJ, Greulich KM, Lee KH, Cram S, Chapman VM, Gray JW (1994). Generation of five high-complexity painting probe libraries from flow-sorted mouse chromosomes. Genomics.

[56] Munné S, Sultan KM, Weier HU, Grifo JA, Cohen J, Rosenwaks Z (1995). Assessment of numeric abnormalities of X, Y, 18, and 16 chromosomes in preimplantation human embryos before transfer. Am. J. Obstet. Gynecol.

[57] Munné S, Weier HU (1996). Simultaneous enumeration of chromosomes 13, 18, 21, X, and Y in interphase cells for preimplantation genetic diagnosis of aneuploidy. Cytogenet. Cell Genet.

[58] Weier HU, Tuton TB, Ito Y, Chu LW, Lu CM, Baumgartner A, Zitzelsberger HF, Weier JF (2006). Molecular cytogenetic characterization of chromosome 9-derived material in a human thyroid cancer cell line. Cytogenet. Genome Res.

[59] Lu CM, Kwan J, Baumgartner A, Weier JF, Wang M, Escudero T, Munné S, Zitzelsberger HF, Weier HU (2009). DNA probe pooling for rapid delineation of chromosomal breakpoints. J. Histochem. Cytochem.

[60] Zeng H, Weier HUG, Kwan J, Wang M, O’Brien B (2011). Data Mining Empowers the Generation of a Novel Class of Chromosome-specific DNA Probes. Journal of Data Mining Genomics Proteomics.

[61] Christensen B, Bryndorf T, Philip J, Lundsteen C, Hansen W (1992). Rapid prenatal diagnosis of trisomy 18 and triploidy in interphase nuclei of uncultured amniocytes by non-radioactive in situ hybridization. Prenat. Diagn.

[62] Waye JS, Durfy SJ, Pinkel D, Kenwrick S, Patterson M, Davies KE, Willard HF (1987). Chromosome-specific alpha satellite DNA from human chromosome 1: hierarchical structure and genomic organization of a polymorphic domain spanning several hundred kilobase pairs of centromeric DNA. Genomics.

[63] Waye JS, Willard HF (1989). Concerted evolution of alpha satellite DNA: evidence for species specificity and a general lack of sequence conservation among alphoid sequences of higher primates. Chromosoma.

[64] Weier HU, Gray JW (1992). A degenerate alpha satellite probe, detecting a centromeric deletion on chromosome 21 in an apparently normal human male, shows limitations of the use of satellite DNA probes for interphase ploidy analysis. Anal. Cell. Pathol.

[65] Baumgartner A, Weier JF, Weier HU (2006). Chromosome-specific DNA repeat probes. J. Histochem. Cytochem.

[66] Lu CM, Kwan J, Weier JF, Baumgartner A, Wang M, Escudero T, Munné S, Weier HU (2009). Rapid mapping of chromosomal breakpoints: from blood to BAC in 20 days. Folia Histochem. Cytobiol.

[67] Cooke HJ, Hindley J (1979). Cloning of human satellite III DNA: different components are on different chromosomes. Nucleic Acids Res.

[68] Cooke HJ, McKay RD (1978). Evolution of a human Y chromosome-specific repeated sequence. Cell.

[69] Cooke HJ, Schmidtke J, Gosden JR (1982). Characterisation of a human Y chromosome repeated sequence and related sequences in higher primates. Chromosoma.

[70] Waye JS, Creeper LA, Willard HF (1987). Organization and evolution of alpha satellite DNA from human chromosome 11. Chromosoma.

[71] Waye JS, England SB, Willard HF (1987). Genomic organization of alpha satellite DNA on human chromosome 7: evidence for two distinct alphoid domains on a single chromosome. Mol. Cell. Biol.

[72] Waye JS, Willard HF (1987). Nucleotide sequence heterogeneity of alpha satellite repetitive DNA: a survey of alphoid sequences from different human chromosomes. Nucleic Acids Res.

[73] Willard HF, Waye JS (1987). Hierarchical order in chromosome-specific human alpha satellite DNA. Trends Genet.

[74] Alexandrov IA, Mashkova TD, Akopian TA, Medvedev LI, Kisselev LL, Mitkevich SP, Yurov YB (1991). Chromosome-specific alpha satellites: two distinct families on human chromosome 18. Genomics.

[75] Weier H-U, Rosette CD, Matsuta M, Zitzelsberger H, Matsuta M, Gray J (1994). Generation of highly specific DNA hybridization probes for chromosome enumeration in human interphase cell nuclei: isolation and enzymatic synthesis of alpha satellite DNA probes for chromosome 10 by primer directed DNA amplification. Methods in Molecular and Cellular Biology.

[76] Cohen D, Chumakov I, Weissenbach J (1993). A first-generation physical map of the human genome. Nature.

[77] Fung J, Munné S Duell T, Weier H-UG (1998). Rapid Cloning of Translocation Breakpoints: from Blood to YAC in 50 Days. J. Biochem. Mol. Biol. Biophys.

[78] Shepherd NS, Pfrogner BD, Coulby JN, Ackerman SL, Vaidyanathan G, Sauer RH, Balkenhol TC, Sternberg N (1994). Preparation and screening of an arrayed human genomic library generated with the P1 cloning system. Proc. Natl. Acad. Sci. U. S. A.

[79] Fung J, Munné S, Garcia J, Kim UJ, Weier HU (1999). Molecular cloning of translocation breakpoints in a case of constitutional translocation t(11,22)(q23,q11) and preparation of probes for preimplantation genetic diagnosis. Reprod. Fertil. Dev.

[80] Shizuya H, Birren B, Kim UJ, Mancino V, Slepak T, Tachiiri Y, Simon M (1992). Cloning and stable maintenance of 300-kilobase-pair fragments of human DNA in Escherichia coli using an F-factor-based vector. Proc. Natl. Acad. Sci. U. S. A.

[81] Osoegawa K, Mammoser AG, Wu C, Frengen E, Zeng C, Catanese JJ, de Jong PJ (2001). A bacterial artificial chromosome library for sequencing the complete human genome. Genome Res.

[82] Altschul SF, Madden TL, Schaffer AA, Zhang J, Zhang Z, Miller W, Lipman DJ (1997). Gapped BLAST and PSI-BLAST: a new generation of protein database search programs. Nucleic Acids Res.

[83] Gray J, Weier Y chromosome specific nucleic acid probe and method
for determining the Y in situ. US Patent number: 5840482. Filing date:
Oct 10, 1990, Issue date: Nov 24th, 1998.

[84] Gray J, Weier Y chromosome specific nucleic acid probe and method
for determining the Y in situ. US Patent number: 6300066, Filing date:
Nov 23, 1998, Issue date: Oct 9, 2001.

[85] Gray J, Weier Y chromosome specific nucleic acid probe and method
for determining the Y in situ. US Patent number: 5888730, Filing date:
Oct 6, 1995, Issue date: Mar 30, 1999.

[86] Lengauer C, Riethman H, Cremer T (1990). Painting of human chromosomes with probes generated from hybrid cell lines by PCR with Alu and L1 primers. Hum. Genet.

[87] Duell T, Nielsen LB, Jones A, Young SG, Weier HU (1998). Construction of two near-kilobase resolution restriction maps of the 5' regulatory region of the human apolipoprotein B gene by quantitative DNA fiber mapping (QDFM). Cytogenet. Cell Genet.

[88] Birnboim HC, Doly J (1979). A rapid alkaline extraction procedure for screening recombinant plasmid DNA. Nucleic Acids Res.

[89] Kwan J, Baumgartner A, Lu CM, Wang M, Weier JF, Zitzelsberger HF, Weier HU (2009). BAC-FISH assays delineate complex chromosomal rearrangements in a case of post-Chernobyl childhood thyroid cancer. Folia Histochem. Cytobiol.

[90] Kent WJ (2002). BLAT--the BLAST-like alignment tool. Genome Res.

[91] Devilee P, Thierry RF, Kievits T, Kolluri R, Hopman AH, Willard HF, Pearson PL, Cornelisse CJ (1988). Detection of chromosome aneuploidy in interphase nuclei from human primary breast tumors using chromosome-specific repetitive DNA probes. Cancer Res.

[92] Bryndorf T, Christensen B, Vad M, Parner J, Carelli MP, Ward BE, Klinger KW, Bang J, Philip J (1996). Prenatal detection of chromosome aneuploidies in uncultured chorionic villus samples by FISH. Am. J. Hum. Genet.

[93] Vorsanova SG, Kolotii AD, Iourov IY, Monakhov VV, Kirillova EA, Soloviev IV, Yurov YB (2005). Evidence for high frequency of chromosomal mosaicism in spontaneous abortions revealed by interphase FISH analysis. J. Histochem. Cytochem.

[94] Weier HU (2008). Cryptic translocations demystified: BAC-FISH assays resolve complex karyotypes in failed human reproduction and cancer. Folia Histochem Cytobiol.

[95] Iourov IY, Liehr T, Vorsanova SG, Yurov YB (2007). Interphase chromosome-specific multicolor banding (ICS-MCB): a new tool for analysis of interphase chromosomes in their integrity. Biomol. Eng.

[96] Zybina TG, Kaufmann P, Frank HG, Freed J, Kadyrov M, Biesterfeld S (2002). Genome multiplication of extravillous trophoblast cells in human placenta in the course of differentiation and invasion into endometrium and myometrium. I. Dynamics of polyploidization. Tsitologiia.

